# Cohort differences in disease and disability in the young-old: findings from the MRC Cognitive Function and Ageing Study (MRC-CFAS)

**DOI:** 10.1186/1471-2458-7-156

**Published:** 2007-07-13

**Authors:** Carol Jagger, Ruth J Matthews, Fiona E Matthews, Nicola A Spiers, Judith Nickson, Eugene S Paykel, Felicia A Huppert, Carol Brayne, the Medical Research Council Cognitive Function and Ageing Study (MRC-CFAS)

**Affiliations:** 1 Leicester Nuffield Research Unit, Department of Health Sciences, University of Leicester, UK; 2 MRC Biostatistics Unit, Institute of Public Health, University of Cambridge, UK; 3 Department of Public Health and Primary Care, Institute of Public Health, Cambridge, UK; 4 Department of Psychiatry, University of Cambridge, Cambridge, UK

## Abstract

**Background:**

Projections of health and social care need are highly sensitive to assumptions about cohort trends in health and disability. We use a repeated population-based cross-sectional study from the Cambridgeshire centre of the UK Medical Research Council Cognitive Function and Ageing Study to investigate trends in the health of the young-old UK population

**Methods:**

Non-overlapping cohorts of men and women aged 65–69 years in 1991/2 (n = 689) and 1996/7 (n = 687) were compared on: self-reported diseases and conditions; self-rated health; mobility limitation; disability by logistic regression and four-year survival by Cox Proportional Hazards Regression models, with adjustments for differences in socio-economic and lifestyle factors.

**Results:**

Survival was similar between cohorts (HR: 0.91, 95% CI: 0.62 to 1.32). There was a significant increase in the number of conditions reported between cohorts, with more participants reporting 3 or more conditions in the new cohort (14.2% vs. 10.1%). When individual conditions were considered, there was a 10% increase in the reporting of arthritis and a significant increase in the reporting of chronic airways obstruction (OR: 1.36, 95% CI: 1.04 to 1.78).

**Conclusion:**

This study provides evidence of rising levels of ill-health, as measured by the prevalence of self-reported chronic conditions, in the newer cohorts of the young-old. Though changes in diagnosis or reporting of disease cannot, as yet, be excluded, to better understand whether our findings reflect real increases in ill-health, investment should be made into improved population-based databases, linking self-report and objective measures of health and function, and including those in long-term care.

## Background

Projections of health and social care need are highly sensitive to assumptions about trends in health and disability, particularly whether life expectancy is increasing slower than healthy life expectancy [1]. The picture from the international literature is mixed, with countries reporting the prevalence of disability to be increasing (Sweden [2]), stagnating (Australia [3], or decreasing (US [4], France [5], Finland [6], Japan [7]) and different evolutions reported for subgroups (men and women in Spain [8] and Denmark [9]) or for mild rather than severe disability (Netherlands [10], Japan [11]). Despite improved levels of functioning in the US, recent cohorts have higher levels of self-reported health problems and diseases [12,13] and more objectively measured markers of disease [14]. These mixed trends have also been reported in the UK by analyses of the General Household Survey for 1977–1994, which reports increased prevalence of chronic illness, but similar levels of self-rated health and ADL disability across cohorts in early old age in England and Wales [15], and by analyses of those aged 75 years and over in the Melton Mowbray Ageing Project where decreases in ADL disability were accompanied by increases in less than good self-rated health [16].

When placed alongside the gains in life expectancy almost universally observed, it appears that all three possible scenarios of compression of morbidity (Fries[17]), expansion of morbidity (Gruenberg[18]) or dynamic equilibrium (Manton[19]) may be occurring. Indeed the latter predicts that reductions in mortality may well be accompanied by increases in chronic disease but that the resulting disability may be less severe, as observed by a number of studies [13].

Thus the evidence from the UK of possibly worsening health at older ages remains unclear, especially as it is expressed through global self-reported measures such as self-rated health and self-reported longstanding illness. As many studies, including the General Household Survey [15] exclude older people in institutional care, and therefore changes in the number and composition of this sector of the population may account for part of the observed trends. Other reasons include change in health behaviours, increase in pathology, earlier diagnosis of conditions, longer survival with conditions, or change in health expectations, and how it may link, to, for example, the marked increase in emergency admissions[20]. Current data, especially outside of the domains of cancer and cardiovascular disease, are too limited to provide answers. Considerable uncertainty therefore remains in this area and is underlined by the range of projections for the future size of the disabled population offered [21,22].

To further elucidate trends in morbidity in the older population in the UK, we analysed representative samples of those aged 65–69 years in 1991/2 and 1996/7, including those in institutions, at a single centre (East Cambridgeshire) of the MRC Cognitive Function and Ageing study (MRC CFAS) to investigate changes in prevalence of disability, self-rated health and a range of self-reported diseases and impairments.

## Methods

A full description of the six-centre MRC CFAS study design can be found elsewhere [23]. This analysis deals with East Cambridgeshire, the only centre to resurvey a repeated cross-section using the same methodology after five years. East Cambridgeshire is a rural area in the East of England which has a similar age profile to the England and Wales national average[24], with a similar proportion retired (National Census data 2001). The proportion of ethnic minorities in this area is small compared with the national average (2.1% vs. 8.7%), and life expectancy is greater than the national average, particularly in women[24].

For the initial screen, random samples of people aged 65 years or over were selected from the National Health Service primary care lists held by the Family Health Service Authority. Identical procedures were repeated in 1996–1997 for the new cohort aged 65–69 years and geographic boundaries remained the same. Individuals provided written consent to participate in the study and all CFAS interviewing was covered by local and multi-centre ethical approval (LREC 95/116 and P93/74). Ascertainment of individuals aged 65–69 years and interviewing was undertaken over a two year time period for each cohort. The exact dates of birth used to generate the sample were

▪ 1991–1992 base cohort

Year 1 1/10/1921 to 30/9/1926 ascertained 30/9/91

Year 2 1/10/1922 to 30/9/1927 ascertained 30/9/92

▪ 1996–1997 new cohort

Year 1 1/10/1926 to 30/9/1931 ascertained 30/9/96

Year 2 1/10/1927 to 30/9/1932 ascertained 30/9/97

All participants were interviewed at their place of residence, including the few in long-term care during 1991–1994 (for the 91/92 cohort) and 1996–1998 (for the new cohort) by trained interviewers who followed a computerised structured interview enquiring about socio-demography, general health (including chronic conditions), cognition, smoking, and physical function. In only 2 cases, one in each cohort, were interviews conducted with a proxy. Death information was provided on all participants who were interviewed from the Office of National Statistics and was complete to 31^st ^December 2004.

Our underlying conceptual framework for defining health was the disablement process, beginning with disease/pathology, through functional limitation to disability[25].

### Disease

Participants were asked if they had ever suffered from heart attack, asthma, chronic bronchitis, arthritis, Parkinson's disease or thyroid problems. Those who had suffered from chronic bronchitis or asthma, (excluding childhood asthma) were classified as having chronic airways obstruction. Diagnostic scales were used for angina and intermittent claudication[26]. Coronary heart disease (CHD) was defined as being present if participants had ever suffered from a heart attack or if angina was indicated by the diagnostic scale. Participants currently receiving treatment for their diabetes or hypertension were coded as having the disease. Participants were classified as having had a stroke if they answered positively to the question "Have you ever had a stroke that required medical attention?" and also reported that the stroke was diagnosed by a GP or specialist.

Cognitive function was assessed using the Mini-Mental State Examination [27]. Missing items were divided according to their nature. 'Don't know', 'no answer' and items which could not be answered due to sensory or mobility problems were recoded to zero. For all other missing items the full score was recoded to missing, unless the individual could be assigned to an MMSE category unambiguously on the basis of completed items. Participants were considered to have moderate to severe cognitive impairment if they scored 0–21 on the MMSE. This cutpoint has been shown to be consistent with a diagnosis of moderate or severe dementia [28].

A score was created of the number of chronic conditions suffered out of 10 possible conditions (stroke, CHD, intermittent claudication, hypertension, arthritis, diabetes, chronic airways obstruction, underactive thyroid, Parkinson's disease and moderate/severe cognitive impairment).

### Functional limitation

Participants were considered to have hearing or eyesight problems if they reported suffering from poor hearing or eyesight (with or without aids) that interfered with day to day living, or the interviewer observed problems that interfered to a marked extent with the interview process. People with negative self-report but interviewer observed sensory limitations were rare (n = 6 with eyesight and n = 10 with hearing limitations).

Mobility limitation was assessed as participants self-report of requiring help with stairs and interviewer rating of permanently chairfast or bedfast.

### Disability

The interviews included items from the modified Townsend activities of daily living scale [23], covering the participant's ability to perform nine activities and tasks, including eight ADL/IADL. The root question was "Are you able to ...?", and response categories were "yes, with no difficulty", "yes, with some difficulty", and "no, needs help". If an activity was not normally undertaken, probing was used to establish whether the participant would be able to undertake the activity in the absence of another person.

Using information on the hierarchy of ADL/IADL [29,30] and based upon the concept of interval of need [31], participants were classified as disabled if they were unable to perform at least one of the following five ADL/IADL without human help: transfer to and from a chair (from interviewer assessment), put on shoes and socks, prepare a hot meal, get around outside, and have a bath or an all-over wash. Those who were able to perform all five activities without human help but who required help with at least one of the two additional instrumental activities of daily living of shopping including carrying heavy bags and heavy housework were classified as having mild disability.

### Statistical methods

Logistic regression models were fitted with disease as the outcome to explore differences between cohorts, adjusting for age and gender (Tables 2 and 3). Ordered logistic regression models were fitted for number of chronic conditions and disability (Table 2) and binary logistic regression models for all other analyses. Models were further adjusted for marital status, education, social class and smoking status to explore whether differences could be explained by socio-demographic differences between cohorts (Table 3). Cox Proportional Hazards models [32] were fitted to compare survival between cohorts, with adjustment for age and gender and further for marital status, education, social class and smoking status. Time was measured from date of interview to date of death, censored at four years from interview.

**Table 2:** Prevalence of chronic conditions and physical limitations in those aged 65–69 years in 1991/92 and those aged 65–69 years in 1996/97

	**Old Cohort (1991/92)**		**New Cohort (1996/97)**		
	**n (%)**	**OR**	**n (%)**	**OR**^a^**(95% CI)**	**p-value**

**Chronic Conditions**					
Number of chronic conditions		1		1.40 (1.15 to 1.71)^b^	0.006
0	195 (28.6)		146 (21.4)		
1	248 (36.4)		253 (37.0)		
2	170 (24.9)		188 (27.5)		
3+	69 (10.1)		97 (14.2)		
Stroke	28 (4.0)	1	33 (4.8)	1.22 (0.72 to 2.05)	0.45
CHD	97 (14.2)	1	96 (14.0)	1.20 (0.87 to 1.65)	0.26
Intermittent claudication	18 (2.6)	1	11 (1.6)	0.61 (0.28 to 1.30)	0.19
Hypertension	174 (25.3)	1	194 (28.3)	1.16 (0.91 to 1.48)	0.21
Arthritis	302 (43.8)	1	367 (53.4)	1.46 (1.18 to 1.82)	< 0.001
Diabetes	31 (4.5)	1	33 (4.8)	1.07 (0.64 to 1.77)	0.80
Chronic airways obstruction	124 (18.0)	1	158 (23.0)	1.36 (1.04 to 1.78)	0.02
Underactive thyroid	32 (4.6)	1	37 (5.4)	1.13 (0.69 to 1.85)	0.62
Parkinsons	3 (0.4)	1	2 (0.3)	0.67 (0.11 to 4.01)	0.65
Cognitive impairment (MMSE 0–21)	30 (4.4)	1	22 (3.2)	0.71 (0.40 to 1.26)	0.24
**Functional Limitations**					
Hearing problems	111 (16.1)	1	90 (13.1)	0.79 (0.58 to 1.08)	0.13
Eyesight problems	40 (5.8)	1	30 (4.4)	0.73 (0.44 to 1.19)	0.19
Mobility limitation	135 (19.6)	1	161 (23.4)	1.25 (0.96 to 1.62)	0.09
DISABILITY		1		1.34 (0.98 to 1.83)^b^	0.06
None	605 (87.9)		579 (84.4)		
Mild	49 (7.1)		63 (9.2)		
Moderate	34 (4.9)		44 (6.4)		
**Self Rated Health**					
Fair/poor vs. Excellent/good	165 (24.0)	1	197 (28.9)	1.28 (1.00 to 1.63)	0.05

^a ^Adjusted for gender and age ^b ^Ordered logistic regression

**Table 3:** Relative risk (95% confidence intervals) of reporting disease by cohort and by socio-demographic factors, adjusted for all other variables in the model

	**Model 1: Number of chronic conditions**	**Model 2: Arthritis**	**Model 3: Chronic airways obstruction**
**Cohort**			
1996/7 vs. 1991/2	1.35 (1.10 to 1.65)	1.42 (1.14 to 1.78)	1.35 (1.02 to 1.77)
**Sociodemographics**			
Sex			
Females *vs*. Males	1.39 (1.12 to 1.72)	1.87 (1.47 to 2.37)	1.07 (0.80 to 1.43)
Age (1 year increase)	1.11 (1.04 to 1.20)	1.08 (1.00 to 1.17)	1.02 (0.93 to 1.12)
Living status			
Lives alone	1	1	1
Lives with spouse	1.16 (0.90 to 1.49)	1.05 (0.79 to 1.39)	1.09 (0.77 to 1.54)
Lives with others	0.91 (0.60 to 1.38)	1.05 (0.66 to 1.67)	0.91 (0.51 to 1.63)
Education			
12+ years	1	1	1
10 – 11 years	0.68 (0.47 to 0.97)	0.77 (0.51 to 1.14)	0.77 (0.46 to 1.27)
0 – 9 years	1.21 (0.88 to 1.67)	1.16 (0.82 to 1.66)	1.00 (0.64 to 1.54)
Social class*			
I & II	1	1	1
III	1.13 (0.89 to 1.44)	1.05 (0.86 to 1.37)	1.20 (0.85 to 1.68)
IV & V	1.30 (0.97 to 1.74)	1.03 (0.75 to 1.43)	1.53 (1.03 to 2.26)
Armed forces/missing	1.23 (0.63 to 2.39)	1.14 (0.52 to 2.48)	1.58 (0.64 to 3.87)
Smoking status			
Never smoker	1	1	1
Current smoker	1.39 (1.09 to 1.76)	1.16 (0.88 to 1.51)	1.51 (1.08 to 2.10)
Ex-smoker	0.93 (0.70 to 1.24)	0.94 (0.68 to 1.30)	1.28 (0.85 to 1.92)

*I & II: Professional & managerial or technical; III: Manual & non-manual; IV & V: Partly & unskilled

## Results

There were 897 individuals aged 65–69 years ascertained on the sample date in the base cohort and 971 in 1996/7. There was a single exclusion due to language ineligibility (first language not English) in the new cohort and 3 in the base cohort with a further 8 in the new cohort not traced. A total of 108 in 1996/7 were sampled from the National Health Service primary care list but never approached because response rates exceeded those projected in the sample size calculation. The response rate improved, from 691 of the 892 (78%) ascertained and eligible individuals in 1991/2 to 688 out of 854 (81%) by 1996/97. The improvement is due to a reduction in refusals, from 175 (20%) in the base cohort to 138(16%) in the new cohort. The study was well known by the time of the new cohort, but protocols for study recruitment, interviewing and training of interviewers were unchanged. A small number (28, 3%, in each cohort) were lost due to death or removal before interview. There were 2 individuals in the base cohort and 1 in the new cohort who cooperated but provided no valid information, and were excluded. For both cohorts, the age and gender distributions of the non-responders and those interviewed were similar (data not shown).

The greater access to education that has occurred was reflected in the higher proportion in the new cohort who had 12 or more years of education (Table 1) though this did not appear to carry through into later occupation as measured by social class. In addition the new cohort were more likely to be living with a spouse and less likely to have smoked (Table 1).

**Table 1:** MRC-CFAS Cambridge 65–69 years cohorts 1991/2 and 1996/7: Sociodemography

	**1991/92 n (%)**	**1996/97 n (%)**
Total	689 (100)	687 (100)
Gender		
Males	343 (49.8)	325 (47.3)
Females	346 (50.2)	362 (52.7)
Age		
64	17 (2.5)	26 (3.8)
65	137 (19.9)	140 (20.4)
66	150 (21.8)	131 (19.1)
67	133 (19.3)	131 (19.1)
68	139 (20.2)	145 (21.1)
69	101 (14.7)	111 (16.2)
70	12 (1.7)	3 (0.4)
Living status		
Alone	155 (22.5)	138 (20.1)
With spouse	475 (68.9)	495 (72.1)
With others	55 (8.0)	51 (7.4)
Institution	4 (0.6)	3 (0.4)
Marital status		
Married/cohabiting	495 (71.8)	522 (76.0)
Single	44 (6.4)	42 (6.1)
Widowed	122 (17.7)	96 (14.0)
Divorced/separated	28 (4.1)	27 (3.9)
Education		
12+ years	72 (10.5)	104 (15.2)
10 – 11 years	151 (22.0)	123 (18.0)
0 – 9 years	465 (67.6)	457 (66.8)
Social class		
I & II	244 (35.4)	204 (27.7)
III	285 (41.4)	302 (44.0)
IV & V	145 (21.0)	166 (24.2)
Armed forces/missing	15 (2.2)	14 (2.0)
Smoking status		
Never smoker	189 (27.5)	241 (35.1)
Ex smoker	333 (48.4)	325 (47.3)
Current smoker	166 (24.1)	121 (17.6)

*I & II: Professional & managerial or technical; III: Manual & non-manual; IV & V: Partly & unskilled

### Survival

Amongst those interviewed in the new cohort, 92.3% (634/687) survived four years after interview, compared with 90.9% (626/689) of those interviewed in 1991/2. There was no significant difference in survival comparing the new with the old cohort (hazard ratio: 0.91, 95% CI: 0.62 to 1.32) after adjustment for age, gender, marital status, education, social class and smoking status or after adjusting for age and gender alone (hazard ratio: 0.85, 95% CI: 0.59 to 1.23).

### Disease, Functioning and disability

There was a significant difference in the number of conditions reported between cohorts (Table 2), with more participants reporting 3 or more conditions in the new cohort (14.2% vs. 10.1% in the old cohort) and fewer reporting no conditions (21.4% vs. 28.6% in the old cohort). When individual conditions were considered, there was a 10 percentage point increase in the reporting of arthritis over the five year period (43.8% vs. 53.4% in the new cohort). The number of people reporting chronic airways obstruction also significantly increased between 1991/92 and 1996/97 (OR: 1.36, 95% CI: 1.04 to 1.78).

The higher prevalence of disease in the newer cohort was mirrored in the borderline significant increase in reporting of poor or fair self-rated health in the new cohort (Table 2). However, no significant differences were observed in functioning or disability between the time periods (Table 2), with small increases in the reporting of disability and mobility limitation, and small decreases in the proportion with hearing and eyesight problems.

Table 3 shows the difference in reporting of disease between 1991/92 and 1996/97, adjusting for sociodemographic factors. The increase in the reporting of number of chronic conditions remained after adjustment for sociodemographic factors (OR: 1.35, 95% CI: 1.10 to 1.65). Significant increases in the reporting of arthritis and chronic airways obstruction also remained after adjustment for sociodemographic factors. Current smokers (compared to never or ex smoker) were more likely to report a greater number of chronic conditions and in particular chronic airways obstruction. Female gender (compared to male) and increasing age were associated with a greater number of chronic conditions and arthritis.

## Discussion

This study provides evidence of rising levels of reported ill-health, as measured by the prevalence of chronic diseases and conditions and, to some extent, in poorer self-rated health, in the newer cohorts of the young-old. These did not appear to be reflected in greater functional limitation or disability although there were small increases in mobility limitation and IADL/ADL disability in the new cohort.

The major contributors to the increase in the number of chronic conditions reported were arthritis and chronic obstructive airways disease, the latter despite lower levels of smoking in the newer cohort. There are few cohort studies of changes in the prevalence of disease and where these exist, findings are equivocal. Newer male cohorts in Sweden were less likely to report no diseases but more likely to report no symptoms [33], whilst recent US cohorts report lower levels of cardiovascular disease but increases in asthma and musculoskeletal problems[34] and Swedish cohorts increased stroke, myocardial infarction and diabetes[35]. Within the US there have been equivocal reporting of trends in the prevalence of arthritis with an increased prevalence in the young-old [36] but decreases for even younger cohorts [34]. Increasing prevalence of arthritis has also been found in Japan [11], associated with an increase in mild disability.

In support that the observed increase in the prevalence of chronic conditions is real, other countries have shown increased prevalence to be also reflected in higher rates of mortality especially from lung cancer, COPD and IHD in Danish, Dutch and Norwegian men[37]. However there may be other reasons for trends, including earlier diagnosis and an increase of reporting of conditions. Newer cohorts may benefit from earlier detection and diagnosis which may be translated into less severe levels of disease and a longer survival from date of diagnosis. We found little evidence of increased survival, perhaps due to low statistical power, but since our data did not include date of diagnosis or severity of disease we could not fully test this hypothesis.

A limitation of our study is that the majority of information on chronic conditions was self-report and therefore our findings, as many of the other studies, may be due to increased awareness and reporting of symptoms in newer cohorts. Although arthritis has already been shown to be an important predictor of moderate to severe disability onset in MRC-CFAS, including the base cohort here [38], the increase in arthritis prevalence in particular may reflect a threshold shift, with those interviewed in 1996/7 reporting arthritis at a lower threshold of pain or impairment, possibly due to increased availability of new treatments. In addition our study had around 4% of each cohort with moderate or severe dementia, where self-report may be problematic. In these cases information was obtained from an informant, with potentially other biases. However previous work has shown that levels of agreement in reports of past and current health problems by informants when compared with participants free of dementia, is good, especially where the informant is co-resident as is likely to be the case for participants with moderate or severe dementia[39].

Our findings of increased ill-health appeared to be accompanied by a small non-significant increase in mobility limitations and ADL disability in later cohorts, since our sample had low statistical power to detect even moderate differences and the five-year interval between cross-sections is fairly short. Cohort differences in impairments, functional limitations and disability have been confined mostly to the US and Scandinavia. Studies from the US have pointed to a decline in both ADL and IADL in recent decades[40], while findings from other studies are less clear. One group of studies report later cohorts to have less hearing impairment [33] and improved physical functioning, the latter generally in mobility and IADL disability rather than in more severe ADL disability, which appears to remain constant [41-43]. The remaining studies point towards later cohorts showing little improvement after socio-demographic changes are taken into account [44,45] or worsening health, including more hearing impairment and worsening levels of objectively measured performance, peak flow and cognition [2]. In the only previous study examining cohort differences in the health of older people in the UK [16], expansion of self-reported ill-health was evident although ADL disability appeared to have improved. As for self-reported information on diseases, increases in disability over time could be due to reductions in the stigma attached to being disabled and therefore later cohorts being more ready to report difficulties with daily life activities.

Although our study is from a rural area in East England, with a predominately white population, and a larger than average life expectancy compared with England and Wales, a strength is that the population is stable. Both cohorts had more than 70% of those interviewed resident in the area for more than 20 years and almost 10% who had been resident for 5 years or less, the new immigrants being broadly comparable in self-rated health, educational level, cognitive function, and level of disability albeit a somewhat higher proportion of new arrivals were classified as social classes 1 & 2 in 1996/7 compared with 1991/2. Comparisons of healthy life expectancy found significant differences between the five centres included in the original MRC CFAS study[46]. However, the prevalence of functional and cognitive impairment were similar compared with the total across all five centres. Furthermore, inclusion of the total population of community-dwelling and institutional residents is a further strength of our study compared with most studies of older people.

The need to determine whether we are living longer healthier lives is imperative for all countries to plan for appropriate levels of health and social care for our expanding older population. There are therefore an increasing numbers of reports in the international literature of cohort or pseudo-cohort studies to answer this important question. That findings are mixed is not unsurprising [47] and may be due in part to differences in birth cohorts, diseases and conditions, severity levels of disability considered, whether objective or subjective measures are used and, for self-rated health, changing expectations. Improvements in the environment and availability of assistive devices and technological aids may also be partly responsible for disability levels remaining constant in the presence of increased disabling disease. Furthermore many studies only cover the population living in the community and some countries, particularly the UK, have seen considerable changes in the delivery of long-term care over the last decade.

The cohorts in our study were born between the two World Wars and are therefore amongst the youngest to be studied to date. Although many of the cohort differences found are not large, in the majority of diseases and conditions, change is in the same direction with increased prevalence of diseases and trends of worsening functioning and disability at milder levels. The lack of evidence of any marked improvement in population health at these ages, casts doubt over the more optimistic scenarios that have been promulgated for future development of health and health services in the UK [1] and adds further support to the hypothesis of expansion of morbidity in the older UK population. It remains to be seen how far trends towards worse self-reported health are attributable to increased surveillance and earlier diagnosis, increased survival with disease, or to cohort differences in the underlying disease-disability processes. Improved population-based databases, linking self-report and objective measures of health and function, and including those in long-term care are required, to understand better these trends and inform appropriate health service and policy responses.

## Conclusion

Our study suggests that the new cohorts of the young-old do not seem to be experiencing longer, healthier lives but report an increased prevalence of chronic conditions, particularly arthritis and chronic airways obstruction.

## Competing interests

The author(s) declare that they have no competing interests.

## Authors' contributions

CJ drafted the manuscript and is guarantor for the paper. RJM undertook the final analysis and helped draft the manuscript. NS undertook the original analysis. FEM contributed to the analysis and helped draft the manuscript. CB conceived the study, participated in its design and coordination and helped draft the manuscript. JN participated in coordination of the study. ESP and FAH participated in the design and coordination of the study and helped draft the manuscript. All authors have seen and agreed the final draft. MRC CFAS is a collaborative study and researchers are acknowledged using corporate authorship.

## Pre-publication history

The pre-publication history for this paper can be accessed here:


